# Cutaneous chemotherapy‐induced radiation recall reaction

**DOI:** 10.1002/ccr3.4306

**Published:** 2021-05-24

**Authors:** Michael J. McKay, Monica Dumbrava, Jeremy Nicholas McKay, Thomas Alexander McKay

**Affiliations:** ^1^ Northern Cancer Service Northwest Cancer Centre Burnie Tas Australia; ^2^ School of Medicine Deakin University Burwood Vic. Australia; ^3^ Faculty of Medicine, Dentistry and Health Sciences Monash University Clayton Vic. Australia

## Abstract

Chemotherapy‐induced radiation recall reactions are rare, commonly affecting skin but can affect internal organs. Treatments include antihistamines and topical steroids and discontinuation of therapy if severe. Rechallenge may not cause recurrence.

## Graphical abstract

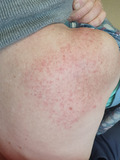

An 80‐year‐old man had metastatic castrate‐resistant prostate cancer, treated with external beam radiotherapy in 2018. A PSA rise was associated with the development of metastatic bone disease in mid‐2019. He was commenced on androgen deprivation therapy with Zoladex, followed by Cosudex, but progressed in bones and was started on Enzalutamide. In March 2020, he received palliative radiotherapy to the right scapula (20 Gy in five fractions, Figure 1A). Because of progressive bone disease, he was commenced on Docetaxel and Denosumab, followed by Cabazitaxel. With further progressive disease, Carboplatin/Paclitaxel was instituted in December 2020: This resulted in the development of a pruritic, morbilliform cutaneous rash which conformed to the site of the previous scapular radiotherapy (Figure 1B). No rash was apparent at the site of previous pelvic radiotherapy. He was prescribed antihistamines and topical hydrocortisone cream, resulting in almost complete clinical resolution over 1 week. The was no return of the rash despite continued chemotherapy.^1^, ^2^

**FIGURE 1:**
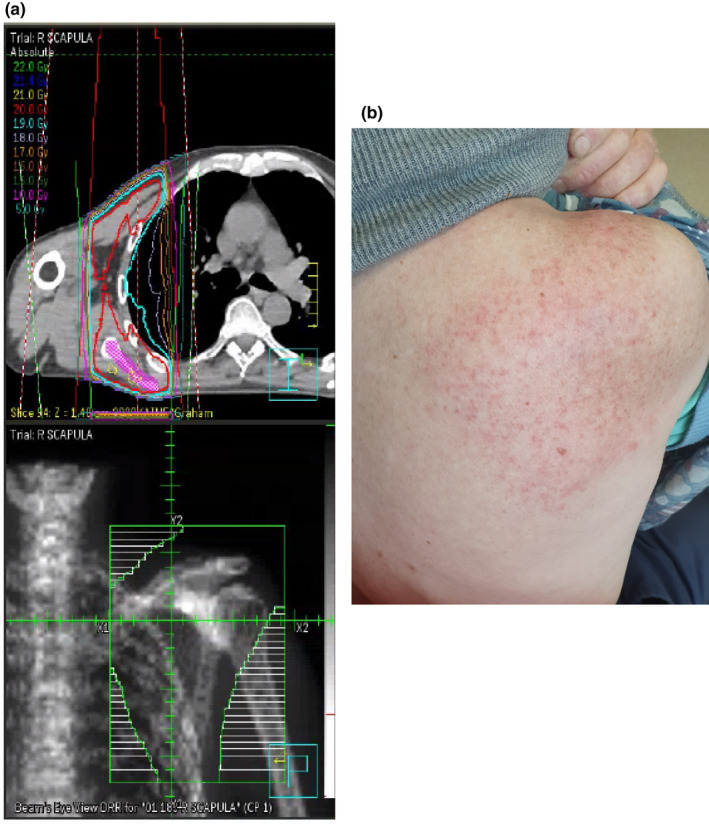
A, (top). Axial computed tomographic image of radiation distribution for right scapular radiotherapy. The target is outlined in pink. A, (bottom). Digitally reconstructed radiograph showing scapular irradiation field. Horizontal bars indicate shielded areas. B, Cutaneous eruption at the site of previous scapular irradiation

## CONFLICT OF INTEREST

None declared.

## ETHICAL APPROVAL

This work has had the approval of the Institutional Ethics Review Board.

## CONSENT STATEMENT

Published with written consent of the patient.

## Data Availability

Data are available by request to the corresponding author.
